# Mediating Role of Treatment Perceptions in the Relationship Between Individual Characteristics and Engagement With a Digital Psychological Intervention for Pediatric Chronic Pain: Secondary Data Analysis

**DOI:** 10.2196/42399

**Published:** 2023-03-06

**Authors:** Rocio de la Vega, Tonya M Palermo

**Affiliations:** 1 Faculty of Psychology University of Malaga Málaga Spain; 2 Instituto Biomedico de Málaga - IBIMA Málaga Spain; 3 Center for Child Health, Behavior and Development Seattle Children’s Research Institute Seattle, WA United States; 4 Department of Anesthesiology and Pain Medicine University of Washington Seattle, WA United States

**Keywords:** treatment adherence, treatment perceptions, mediators, pediatric pain, psychological intervention, digital health, treatment, intervention, engagement, self-management, psychological

## Abstract

**Background:**

Engagement predicts benefits from self-managed treatments. However, engagement is an important concern in digital interventions, with over 50% of patients being nonadherent to interventions in chronic conditions such as chronic pain. Little is known about the individual characteristics that contribute to engagement with a digital self-management treatment.

**Objective:**

This study tested the mediating role of treatment perceptions (difficulty and helpfulness) in the association between individual baseline characteristics (treatment expectancies and readiness to change) and treatment engagement (online and offline) with a digital psychological intervention for adolescents with chronic pain.

**Methods:**

A secondary data analysis of a single-arm trial of Web-based Management of Adolescent Pain, a self-guided internet intervention developed for the management of chronic pain in adolescents, was conducted. Survey data were collected at baseline (T1), midtreatment (ie, 4 weeks after the treatment started; T2), and post treatment (T3). Online engagement was assessed using back-end information on the number of days adolescents accessed the treatment website, while the offline engagement was assessed with the reported frequency of use of skills (ie, pain management strategies) learned at the end of the treatment. Four parallel multiple mediator linear regression models, using ordinary least square regression incorporating the variables were tested.

**Results:**

In total, 85 adolescents with chronic pain (12-17 years old, 77% female) participated. Several mediation models were significant in predicting online engagement. A significant indirect effect was found for the path expectancies–helpfulness–online engagement (effect 0.125; SE 0.098; 95% CI 0.013-0.389) and for the path precontemplation–helpfulness–online engagement (effect −1.027; SE 0.650; 95% CI −2.518 to −0.054). Fourteen percent of the variance of online engagement was explained by the model including expectancies as a predictor (*F*_3_=3.521; *P*<.05), whereas 15% was explained by the model where readiness to change was the predictor (*F*_3_=3.934; *P*<.05). Offline engagement was partially explained in the model including readiness to change as the predictor but with marginal significance (*F*_3_=2.719; *R*^2^=0.111; *P*=.05).

**Conclusions:**

Treatment perception, specifically, perceived helpfulness, was a mediator of the pathway between both treatment expectancies and readiness to change and online engagement with a digital psychological intervention for chronic pain. Assessing these variables at baseline and midtreatment may help to determine the risk of nonadherence. Further work is needed to confirm these mediation pathways in larger samples.

**Trial Registration:**

ClinicalTrials.gov NCT04043962; https://clinicaltrials.gov/ct2/show/NCT04043962

## Introduction

Chronic pain is a burden for patients and their families; it has been declared a global health priority [1]. Multidisciplinary treatments have shown success in improving patient function and quality of life; however, evidence-based interventions are not widely available. The difficulty accessing pain clinics and the long waitlist times [2] call for finding new and complementary approaches to address this problem. The National Pain Strategy [3] recommends promoting and enabling self-management of pain through technology. With the development of technology and digital platforms, self-managed digital interventions are a rising therapeutic approach that can help bring evidence-based interventions to the user [4,5]. By translating validated in-person therapeutic approaches into digital interventions, the number of people with chronic pain that can receive such interventions can be dramatically increased by eliminating important barriers of access such as limited economic resources, distance from the clinics, waitlists, or limited mobility. This may allow for a significant improvement of access to specialized care and help reduce disparities. A meta-analysis on technology-delivered psychological interventions has shown some efficacy across chronic pain conditions including significantly reducing headache intensity and producing satisfaction with treatment for mixed pain conditions [5]. However, there is considerable variability in treatment engagement and treatment response. A call has been made to understand the moderators and mediators involved in predicting benefits from psychological treatments [6], in particular, to determine factors that could be targeted in different or alternative interventions that could be offered to those at risk of low treatment response [7].

Digital health approaches offer several advantages when testing for potential treatment mechanisms, such as exposing all participants to the same intervention, ensuring treatment fidelity, and collecting data prospectively at different points during the intervention in real-time, allowing for better testing of treatment mediators and processes.

On the other hand, patients following self-managed interventions are not closely monitored by their therapists, and engagement with treatment is self-motivated. Consequently, a strong candidate for mediating treatment efficacy is patient engagement, as it has been shown to be associated with treatment outcomes [8]. This is an important concern in digital interventions, with over 50% of patients being nonadherent to interventions in chronic conditions such as chronic pain [9]. Expert consensus on priority topics for future research in engagement with behavior change interventions suggests focusing on “effective engagement” instead of just “more engagement,” that is, sufficient engagement with the intervention in a way that the intended outcomes are achieved [10]. It is also recommended to distinguish between engagement with the digital intervention (online engagement) and engagement with the behavior changes taught by the intervention (offline engagement). For example, a higher use of the digital intervention (eg, more interaction and more hours spent using it) has been traditionally considered a good measure of engagement; however, it is possible that users disengage with the intervention on the web because they have already learnt the skills to change behaviors and continue to engage with those changes offline. Alternatively, users may keep engaging with the intervention digitally because they are struggling to learn the new behavior and are not able to engage with it offline.

One of the factors impacting treatment engagement is motivation. According to the Motivational Model for Pain Self-Management [11], motivation for treatment engagement is often low in people with chronic pain. It varies as a function of the perceived importance of engaging with treatment (eg, expectancies about the treatment benefits). In this study, we consider motivation to engage with the treatment in 2 ways. On the one hand, as expectancies about the treatment benefits and on the other as pretreatment readiness to change (proposed by the Transtheoretical Model of Change [12]), a concept that has been extensively studied in smoking cessation, diabetes treatment adherence, and other fields since the 1980s. In adults, high pretreatment readiness to change is associated with improvements in pain and psychological functioning after behavioral treatment [13-16]. In children, Simons et al [17] found that the strongest predictor of nonresponse after an intensive pain rehabilitation program was low readiness to change. Fortunately, readiness to change is not a trait, but a state, and it has been shown to increase after multidisciplinary pain treatment (for both children and their parents) [18].

Finally, to integrate the effects of those baseline characteristics with treatment processes, we took as a referent the Behavior Change Model for Internet Interventions [19]. This model aims to integrate treatment processes for remotely delivered interventions; one focus of this model is the perceptions patients have of the treatment, as an element that can influence engagement with the intervention. These perceptions can include, for instance, how difficult the treatment is to follow and how helpful the treatment is for coping with symptoms.

In summary, despite engagement being a clearly important variable to consider and understand, little is known about the individual characteristics that contribute to engagement with a digital self-management treatment, especially in adolescents. It is important to better understand how some baseline characteristics are associated with motivation to follow the treatment and with perceptions about it, and whether or not this influences the level of engagement with the intervention. Consequently, the main aim of this study is to examine baseline characteristics (ie, readiness to change and expectancies) as predictors of treatment perceptions (ie, helpfulness of the treatment and difficulty following the treatment) and adherence to the treatment (both online and offline).

We hypothesized that both online (ie, number of days accessing the treatment website) and offline (ie, reported frequency of use of skills learned at the end of the treatment) engagement will be significantly and independently predicted by (1) low readiness to change, as evidenced by precontemplation scores (negative association) and (2) treatment expectancies (positive association), and they will be mediated by treatment perceptions: helpfulness (positive association) and difficulty following the treatment (negative association). In addition, we expect that individual characteristics will be significantly associated with treatment perceptions, specifically, higher expectancies will be positively associated with higher perceived helpfulness and lower difficulty following treatment recommendations, whereas precontemplation scores will have a negative association.

## Methods

### Procedures

In order to address our aims, we examined treatment process variables during the participation in a single-arm trial of Web-based Management of Adolescent Pain (WebMAP), a self-guided internet intervention developed for the management of chronic pain in adolescents [20]. A single-arm clinical trial design was chosen to study treatment processes because the efficacy of the intervention has already been proven for several outcome variables [20-24]. Another article has been published about baseline sleep as a predictor of treatment response using data from the same trial [25]; however, the variables studied in the present report and the aims are different.

### Ethics Approval

The trial is registered on ClinicalTrials.gov (NCT04043962). The primary study and subsequent modifications were approved by the institutional review board at Seattle Children’s Research Institute.

During the study period, participating adolescents had access to Web-based Management of Adolescent Pain (WebMAP), an interactive web-based intervention covering different aspects related to pain self-management and well-being, specifically education, stress and negative emotions, deep breathing and relaxation, coping skills at school, cognitive skills, sleep and lifestyle, staying active, and relapse prevention. There are 8 treatment modules assigned at a pace of completing 1 module per week. Data collection took place using the Research Electronic Data Capture (REDCap, Vanderbilt University [26]) secure system, and in this report, we use data from baseline (T1), midtreatment (ie, 4 weeks after the treatment started; T2), and post treatment (T3). Full details can be found in the main outcomes study description [25].

### Participants

Inclusion criteria were (1) being 12-17 years old, (2) diagnosed with a primary pain disorder by a specialty physician in one of the participating clinics, (3) experiencing pain for at least 3 months, and (4) having access to the internet. In total, 85 adolescents with chronic pain participated in the study (12-17 years old). They were recruited from 2 multidisciplinary pain clinics (one specialized in headache and the other in complex chronic pain) at Seattle Children’s Hospital from November 2018 to February 2020. The exclusion criteria were (1) presenting another serious health condition (eg, cancer), (2) not speaking English, (3) having active psychosis or suicidal ideation, and (4) having a diagnosis of sleep apnea or narcolepsy (due to the aims of the main study).

### Measures

#### Demographic and Clinical Characteristics

Adolescent’s age, sex, race and ethnicity, annual household income, and parents’ education were reported by the parents. Pain characteristics (bodily location, intensity, and frequency over the past 3 months) were reported by the adolescents. A 0 to 10 numerical rating scale [27] was used to assess average pain intensity.

#### Baseline Characteristics (T1, Pretreatment)

##### Treatment Expectancies

Treatment expectancies were measured with the Treatment Expectancies Questionnaire, which assesses how participants think a treatment that may help adolescents with chronic pain. The 10-item measure assesses how likely it is that a chronic pain internet program may be useful for adolescents with chronic pain and for the management of chronic pain in different ways (eg, having less pain and making better lifestyle choices). Items are rated on a 5-point Likert scale (1=“not at all likely” to 5=“extremely likely”). Total scores have a possible range of 10-50, with higher scores indicating higher expectancies. This measure has been used in a previous study with pediatric chronic pain populations [28]. Cronbach *α* was .92 in this study.

##### Readiness to Adopt a Self-management Approach to Pain

Readiness to change was measured with the 30-item Pain Stages of Change Questionnaire – Adolescent version (PSOCQ-A [29]), which assesses how ready an individual is to practice self-management of pain. The items of the PSOCQ-A assess to what extent someone is ready to act (eg, “I have been thinking that the way I cope with my pain could get better” and “I am developing new ways to cope with my pain.”). Items are rated on a 5-point Likert scale (1=“strongly disagree” to 5=“strongly agree”). The PSOCQ-A consists of 4 factors: precontemplation (in which the individual has little interest in changing their behaviors), contemplation (in which the individual is thinking about the behavioral change but is not likely to change soon), action (in which the individual is considering behavioral change and is likely to engage in change within a month), and maintenance (in which the individual is trying to maintain their behavioral changes). Each factor has a possible range of 1-5, with higher scores indicating a greater likelihood of being in that stage of behavior change. We used the precontemplation scale for this study, as it indicates a low readiness to change, which we identified as important in predicting treatment response. Cronbach *α* for this scale was .75.

#### Treatment Perceptions (T2, Midtreatment)

##### Treatment Helpfulness

Participants were asked to rate on a 4-point Likert scale “How positively is the treatment affecting you?” (0=“did not affect me at all” to 3= “completely”).

##### Treatment Difficulty

Participants were asked to rate on an 11-point Likert scale “How difficult are the treatment strategies for you to use?” (0=“extremely easy” to 10=“impossible to do”).

#### Treatment Engagement (T3, Immediate Post-treatment)

##### Online Engagement

Online engagement with the treatment was assessed using back-end information from the treatment website. Specifically, the number of modules completed and the number of days adolescents accessed the treatment website were recorded.

##### Offline Engagement

Offline engagement was assessed with the reported frequency of use of skills learned at the end of the treatment. Participants were first presented with a list of all the skills taught in the treatment and asked to rate, on a 5-point Likert scale “How often are you currently using them?” (0=“never” to 4=“every day”).

### Statistical Analysis

#### Power

As a secondary data analysis study, the sample size was determined by the number of participants (N=85) in the primary trial. That sample size is estimated to be enough to test all the paths of the mediation models proposed for this study, following the recommendations of Hayes and Rookwood [30] of including at least 10 participants per each path to be tested.

#### Data Analysis Plan

In order to test all the hypotheses, we first computed Pearson correlations between all the relevant variables: precontemplation scores, treatment expectancies, perceptions about the treatment, online engagement, and offline engagement.

We then planned to integrate all the significant associations into 4 parallel multiple mediator linear regression models, using ordinary least-squares regression (model #4 in PROCESS [30]) to test for significant paths. Four models were used instead of 1 large comprehensive model due to the limited sample size that was available to conduct alternative analyses. We used treatment expectancies as the predictor (X) in models 1 and 2, precontemplation scores as the predictor in models 3 and 4, and engagement as the dependent variable (Y): online engagement in models 1 and 3 and offline engagement in models 2 and 4. The mediators (M) for all models were treatment perceptions: helpfulness and difficulty. All data analyses were conducted using SPSS (version 26; IBM Corp) for Mac [31] and the PROCESS 3.5 macro (Hayes et al) [30].

## Results

### Participant Characteristics

Participants were mostly female (n=65, 77%) and 13 (15%) were Hispanic, with a mean age of 15 (SD 1.5) years. Parents were well educated and with a medium to high income. Regarding the clinical characteristics, average pain intensity in the sample was 5.7 (SD 1.7) out of 10, average number of pain locations was 4 (SD 2.3), and the frequency of pain was daily for over half of the sample (n=57, 67%). See Table 1 for details.

Adolescents completed an average of 7 (SD 2.5) of the 8 modules of the treatment, with 51 (60%) of them completing at least 7 modules. Given the low variability, we decided to use the number of days they logged into the treatment program to compute the amount of online engagement. The two variables (ie, modules completed and days logging in) are moderately correlated (*r*=0.54; *P*<.001).

**Table 1:** Baseline characteristics of the sample (N=85).

Characteristics			Values
**Demographic characteristics**			
	Age (years), mean (SD)		15.5 (1.5)
	Sex (female), n (%)		65 (77)
	**Ethnicity, n (%)**		
		Hispanic or Latino	13 (15)
	**Race, n (%)**		
		American Indian/Alaska Native	5 (6)
		Asian	7 (8)
		Black	4 (4.6)
		Latin American	7 (8)
		White	77 (91)
		More than one race	16 (18)
	**Annual household income (US $), n (%)**		
		<49,999	24 (28)
		50,000-99,999	25 (29)
		>100,000	35 (41)
		Not reported	1 (1)
	**Parents’ education, n (%)**		
		High school or less	4 (5)
		College or vocational school	60 (70)
		Graduate or professional school	20 (24)
		Not reported	1 (1)
**Clinical characteristics**			
	Usual pain severity (0-10 NRS^a^), mean (SD)		5.7 (1.7)
	**Pain frequency (past 3 months), mean (SD)**		
		Daily	57 (67)
		Weekly	22 (26)
		Monthly	4 (5)
		Not reported	2 (2)
	Pain locations (0-9), mean (SD)		4 (2.3)

^a^NRS: numerical rating scale.

### Bivariate Associations Among Treatment Expectancies, Readiness to Change, Treatment Perceptions, and Engagement With the Intervention

Participants’ expectancies were moderately high on average (30.4 out of 50, SD 7.1). Their precontemplation scores were 3.2 (SD 0.9) over 5, indicating a low readiness to change. Treatment perceptions were mixed, as evidenced by a moderate perceived helpfulness score (1.8 on a 0-3 scale; SD 0.9) but also a moderate perceived difficulty score (6.9 out of 10; SD 2.3). Online engagement was adequate, with an average of 12.9 days (over 8 weeks) logging into the treatment, although there was high variability (SD 12.2). Offline engagement was high, with a mean score of 3.1 on a 0-4 scale (SD 0.9).

As shown in Table 2, higher expectancies (T1) were significantly associated with lower precontemplation scores (ie, higher readiness to change; T1), higher perceived helpfulness of the treatment (T2), and higher offline engagement (T3). Offline engagement was also positively associated with the perceived helpfulness of the treatment (T2). Perceived helpfulness was, on the other hand, negatively associated with baseline precontemplation. Finally, online engagement (T3) was only associated with perceived difficulty of the treatment (T2), that is, higher perceived difficulty was associated with more online engagement.

**Table 2:** Descriptive statistics and correlations between treatment variables.

Variables	Mean (SD)	Expectancies	Precontemplation	Difficulty of treatment	Helpfulness of treatment	Online engagement	Offline engagement
Expectancies	30.4 (7.1)	—^a^					
Precontemplation	3.2 (0.9)	−0.30^b^	—				
Difficulty of treatment	6.9 (2.3)	0.02	0.02	—			
Helpfulness of treatment	1.8 (0.9)	0.32^b^	−0.28^c^	−0.02	—		
Online engagement	12.9 (12.2)	0.02	0.08	0.26^c^	0.22	—	
Offline engagement	3.1 (0.9)	0.24^c^	−0.11	0.19	0.27^c^	0.08	—

^a^Not available.

^b^Pearson correlations significant at *P*<.01.

^c^Pearson correlations significant at *P*<.05.

### Mediation Models

#### Model 1: Mediation Model With Expectancies Predicting Online Engagement

**Figure 1:**
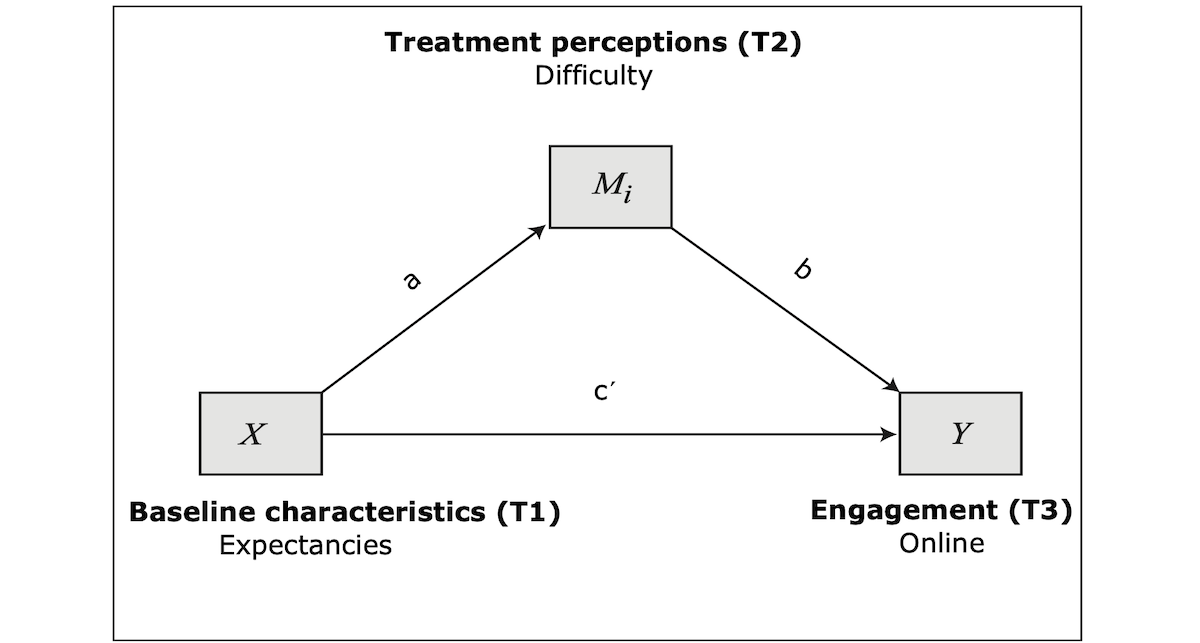
Model 1: mediation model with expectancies predicting online engagement.

The first model proposes that expectancies would impact online engagement directly and treatment mediators which would, in turn, impact online engagement (see Figure 1).

The full regression model explained 14% of the variance of online engagement (*F*_3_=3.521; *R*^2^=0.14; *P*<.05); however, only some paths of the model were significant. Specifically, as hypothesized, higher treatment expectancies predicted higher perceived helpfulness (path *a*2: B=0.039; SE 0.014; *P*<.05; 95% CI 0.011-0.06), but they did not significantly predict perceived difficulty. Direct effects (Table 3) of treatment perceptions were also significant: higher perceived helpfulness led to more online engagement (path *b*2: B=3.948; SE 1.718; *P*<.05; 95% CI 0.516-7.380), as predicted, and, contrary to the hypotheses, higher perceived difficulty also predicted more online engagement (path *b*1: B=1.468; SE 0.615; *P*<.05; 95% CI 0.240-2.696). The direct effect from expectancies to online engagement was not significant. Finally, regarding the indirect effects (Table 4), the path expectancies–difficulty–online engagement was nonsignificant, as the 95% bootstrap CIs contained zero. On the other hand, the path expectancies–helpfulness–online engagement is statistically significant as the CI did not contain zero (effect=0.125, SE 0.098; 95% CI 0.013-0.389).

**Table 3:** Summary of model 1^a^: direct effects.

Direct effects	Path	B	SE	*P* value	95% CI
Expectancies to difficulty	*a*1	0.004	0.038	.923	−0.073 to 0.080
Expectancies to helpfulness	*a*2	0.039	0.014	.007	0.011 to 0.066
Difficulty to online engagement	*b*1	1.468	0.615	.019	0.240 to 2.696
Helpfulness to online engagement	*b*2	3.948	1.718	.025	0.516 to 7.380
Expectancies to online engagement	*c*’	−0.059	0.205	.773	−0.468 to 0.349

^a^*R*=0.374; *R*^2^=0.140; *F*_3,65_=3.521; *P*=.02.

**Table 4:** Summary of model 1: indirect effects.

Indirect effects	Boot^a^ effect	Boot SE	Boot LLCI^b^	Boot ULCI^c^
Expectancies to difficulty to online engagement	0.006	0.069	−0.134	0.147
Expectancies to helpfulness to online engagement	0.152	0.098	0.013	0.389

^a^Boot: statistics for the indirect effects are the result of the bootstrapping method.

^b^LLCI: lower limit 5% CI.

^c^ULCI: upper limit 95% CI.

#### Model 2: Mediation Model With Expectancies Predicting Offline Engagement

**Figure 2:**
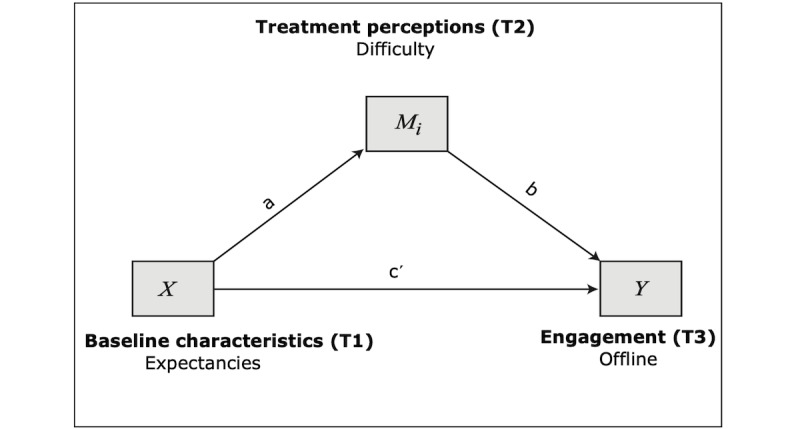
Model 2: mediation model with expectancies predicting offline engagement.

The second model proposes that expectancies would impact offline engagement directly and treatment mediators which would, in turn, impact offline engagement (see Figure 2). That is, the variables are similar to model 1, with the exception of the outcome (offline engagement).

The full regression model was not significant (*P*=.39). However, some of the paths were significant. Similar to model 1, as hypothesized, higher treatment expectancies predicted higher perceived helpfulness (path *a*2: B=0.032; SE 0.014; *P*<.05; 95% CI 0.004-0.060), but they did not significantly predict perceived difficulty. Direct effects (Table 5) of expectancies or treatment perceptions on offline engagement were not significant in this model. Finally, indirect effects (Table 6) were not significant.

**Table 5:** Summary of model 2^a^: direct effects.

Direct effects	Path	B	SE	*P* value	95% CI
Expectancies to difficulty	*a*1	0.013	0.039	.743	−0.066 to 0.092
Expectancies to helpfulness	*a*2	0.032	0.014	.025	0.004 to 0.060
Difficulty to offline engagement	*b*1	0.002	0.052	.976	−0.103 to 0.106
Helpfulness to offline engagement	*b*2	0.212	0.147	.153	−0.081 to 0.505
Expectancies to offline engagement	*c*’	0.016	0.164	.325	−0.017 to 0.049

^a^*R*=0.219; *R*_2_=0.048; *F*_3,61_=1.025; *P*=.39.

**Table 6:** Summary of model 2: indirect effects.

Indirect effects	Boot^a^ effect	Boot SE	Boot LLCI^b^	Boot ULCI^c^
Expectancies to difficulty to offline engagement	0.000	0.003	−0.011	0.004
Expectancies to helpfulness to offline engagement	0.007	0.007	−0.003	0.026

^a^Boot: statistics for the indirect effects are the result of the bootstrapping method.

^b^LLCI: lower limit 5% CI.

^c^ULCI: upper limit 95% CI.

#### Model 3: Mediation Model With Precontemplation Predicting Online Engagement

**Figure 3:**
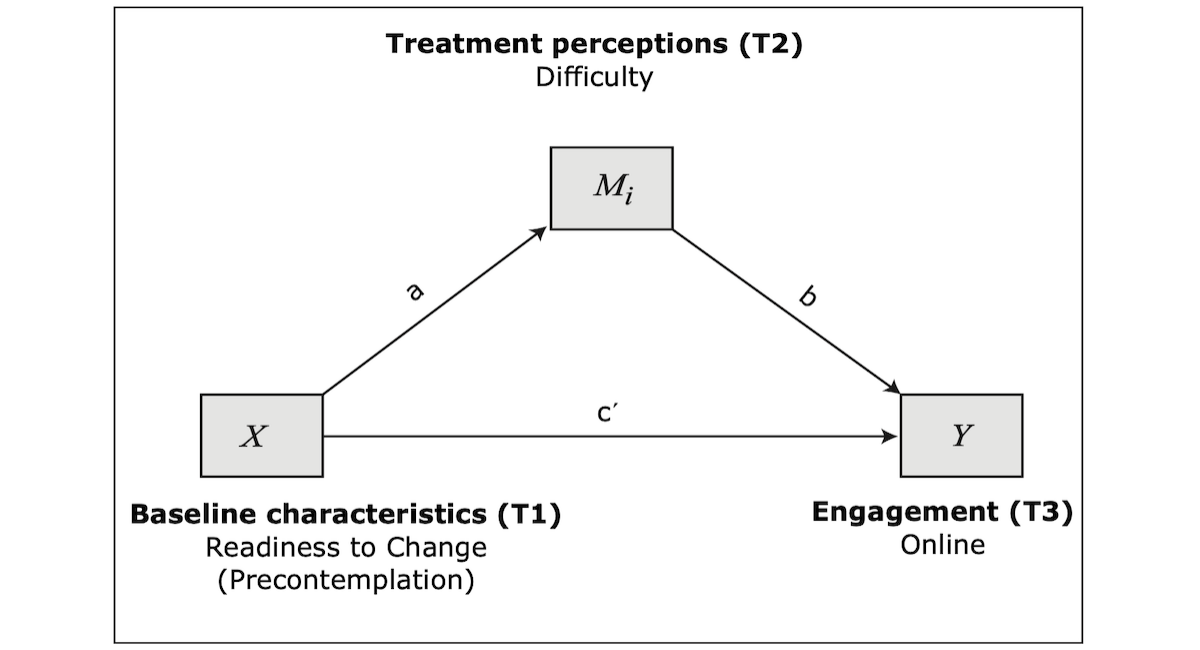
Model 3: mediation model with precontemplation predicting online engagement.

The third model proposes that readiness to change, precontemplation scores, specifically, would impact online engagement directly and treatment mediators which would, in turn, impact online engagement (see Figure 3). That is, the variables are similar to model 1, with the exception of the predictor.

The full regression model explained 15% of the variance of online engagement (*F*_3_=3.934; *R*^2^=0.154; *P*<.05); however, only some paths of the model were significant. Specifically, as hypothesized, higher precontemplation scores predicted lower perceived helpfulness (path *a*2: B=−0.254; SE 0.108; *P*<.05; 95% CI −0.468 to −0.039), but they did not significantly predict perceived difficulty. Direct effects of treatment perceptions were also significant: higher perceived helpfulness led to more online engagement (path *b*2: B=4.047; SE 1.678; *P*<.05; 95% CI 0.696-7.397), as predicted, and, contrary to the hypotheses, higher perceived difficulty also predicted more online engagement (path *b*1: B=1.457; SE 0.610; *P*<.05; 95% CI 0.240-2.675). The direct effect (Table 7) from precontemplation to online engagement was not significant. Finally, regarding the indirect effects (Table 8), the path precontemplation–difficulty–online engagement was nonsignificant, as the 95% bootstrap CIs contained zero. On the other hand, the path precontemplation–helpfulness–online engagement is statistically significant as the CI did not contain zero (effect=−1.027; SE 0.650; 95% CI −2.518 to −0.054).

**Table 7:** Summary of model 3^a^: direct effects.

Direct effects	Path	B	SE	*P* value	95% CI
Precontemplation to difficulty	*a*1	0.012	0.296	.968	−0.579 to 0.603
Precontemplation to helpfulness	*a*2	−0.254	0.108	.021	−0.468 to −0.039
Difficulty to online engagement	*b*1	1.457	0.610	.020	0.240 to 2.675
Helpfulness to online engagement	*b*2	4.047	1.678	.019	0.696 to 7.397
Precontemplation to online engagement	*c*’	1.276	1.572	.420	−1.862 to 4.415

^a^*R*=0.392; *R*^2^=0.154; *F*_3,65_=3.934; *P*=.01.

**Table 8:** Summary of model 3: indirect effects.

Indirect effects	Boot^a^ Effect	Boot SE	Boot LLCI^b^	Boot ULCI^c^
Precontemplation to difficulty to online engagement	0.017	0.437	−0.793	0.964
Precontemplation to helpfulness to online engagement	−1.027	0.650	−2.518	−0.054

^a^Boot: statistics for the indirect effects are the result of the bootstrapping method.

^b^LLCI: lower limit 5% CI.

^c^ULCI: upper limit 95% CI.

#### Model 4: Mediation Model With Precontemplation Predicting Offline Engagement

**Figure 4:**
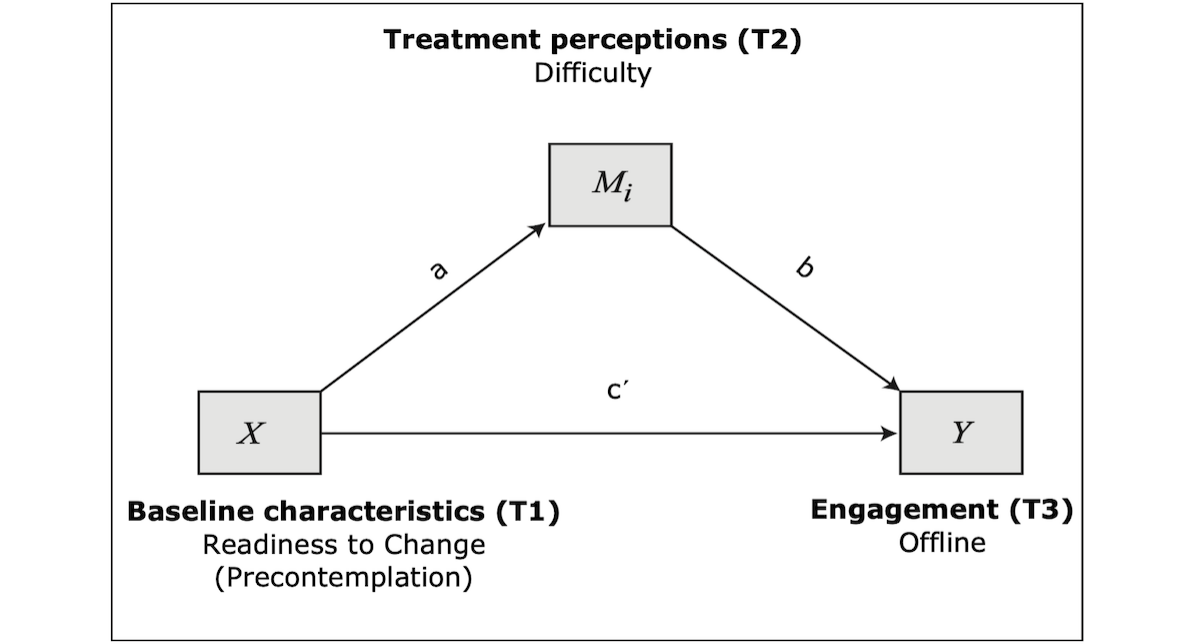
Model 4: mediation model with precontemplation predicting offline engagement.

The fourth and final model proposes that readiness to change would impact offline engagement directly and treatment mediators which would, in turn, impact offline engagement (see Figure 4). That is, the variables are similar to model 2 with the exception of the predictor (readiness to change).

The full regression model was marginally significant (*P*=.05), explaining 11% of the variance of offline engagement (*F*_3_=2.719; *R*^2^=0.111). Additionally, some of the paths were significant. Similar to model 3, as hypothesized, lower readiness to change (ie, higher precontemplation scores) predicted higher perceived helpfulness (path *a*2: B=−0.239; SE 0.106; *P*<.05; 95% CI −0.449 to 0.028), but it did not significantly predict perceived difficulty. Direct effects (Table 9) on offline engagement were only significant for perceived helpfulness in this model (path *b*2: B=0.213; SE 0.104; *P*<.05; 95% CI 0.006-0.420). Finally, indirect effects (Table 10) were not significant.

**Table 9:** Summary of model 4^a^: direct effects.

Direct effects	Path	B	SE	*P* value	95% CI
Precontemplation to difficulty	*a*1	−0.073	.277	.793	−0.625 to 0.479
Precontemplation to helpfulness	*a*2	−0.239	.106	.027	−0.449 to −0.028
Difficulty to offline engagement	*b*1	0.063	.040	.118	−0.016 to 0.142
Helpfulness to offline engagement	*b*2	0.213	.104	.044	0.006 to 0.420
Precontemplation to offline engagement	*c*’	−0.100	.093	.287	−0.285 to 0.086

^a^*R*=0.333; *R*^2^=0.111; *F*_3,65_=2.710; *P*=.05.

**Table 10:** Summary of model 4: indirect effects.

Indirect effects	Boot^a^ Effect	Boot SE	Boot LLCI^b^	Boot ULCI^c^
Precontemplation to difficulty to offline engagement	−0.005	0.024	−0.066	0.035
Precontemplation to helpfulness to offline engagement	−0.051	0.035	−0.131	0.002

^a^Boot: statistics for the indirect effects are the result of the bootstrapping method.

^b^LLCI: lower limit 5% CI.

^c^ULCI: upper limit 95% CI.

## Discussion

### Principal Results

This secondary data analysis of a single-arm trial of digital psychological intervention for adolescents with chronic pain evaluated individual baseline and psychological variables that predict engagement with the intervention program. For the first time, the concepts of online versus offline engagement were examined separately.

In order to better understand how those variables are related to each other, we built 4 mediation models to test for their interactions, all of them using individual characteristics (T1) as predictors, treatment perceptions (T2) as mediators, and engagement (T3) as outcomes. Two models were built with online engagement as the outcome. Both models predicted a small but significant amount of variance (perceived helpfulness was a mediator of the pathway between the predictors and online engagement). Two models, on the other hand, had offline engagement as the outcome, and these models were not significant.

Specifically, a significant indirect effect was found for the path expectancies–helpfulness–online engagement and for the path precontemplation–helpfulness–online engagement with similar variance of online engagement explained by the model when expectancies was a predictor as when readiness to change was the predictor. Offline engagement was partially explained in the model including readiness to change as the predictor, but with marginal significance.

### Comparison With Prior Work

As proposed by the Motivational Model for Pain Self-Management [11], our results show that readiness to adopt a self-management approach for pain management is key in engagement with a digital psychological intervention for adolescent chronic pain. Prior studies have also shown that individuals with high precontemplation scores tend to believe that pain management is the responsibility of the health care professionals (or of their parents, in the case of adolescents) [29].

Specifically, we found that readiness to change influences treatment perceptions (ie, lower readiness is related with poorer perceptions) and that this, in turn, had an impact on engagement. Similar results were observed in relation to the association between expectancies and engagement. The Motivational Model for Pain Self-Management [11] suggests that pretreatment interventions could be administered before starting treatment, as a “pre-habilitation” intervention, in order to ensure the patient is ready to adopt the treatment recommendations. Hence, assessing the stage of readiness to change and determining whether patients with low versus high levels approach (and perceive) the treatment differently is a relevant aspect to consider when deciding when patients should receive a self-administered treatment. Pretreatment sessions (eg, motivational interviews and psychoeducation) could be implemented to increase readiness to change. For instance, conducting motivational interviewing can help the patient feel heard and validated and to overcome ambivalence about starting the treatment by focusing on their specific goals. Additionally, therapists can provide education on the bio-psycho-social dimensions of pain and how the way patients behave, think, and experience emotions has an impact on subsequent pain and associated symptoms. This may help the patients to better understand how having an active role following an intervention of this kind may be helpful for their pain and increase their expectancies and their willingness or readiness to engage with it.

Focusing on the selected mediators, we found that treatment perceptions, specifically perceived helpfulness of the treatment, predicted engagement. Assessing such perceptions midtreatment, which could be done on the web, using the website or app used to deliver the digital treatment, could be used to trigger warnings for the therapist in charge (in the case of supervised interventions) or to trigger booster modules (in the case of stand-alone interventions). This may also be a criterion for stepping up care to involve human support, especially when the treatment is perceived as difficult to follow, as coaching guidance has been shown to increase adherence to digital treatments in a recent meta-analysis [32]. This could help integrate stand-alone treatments into a stepped model of care, that is, if midtreatment assessment shows high perceived difficulty, the patient may need to move to a supervised intervention with a coach that can review the exercises, discuss difficulties found, and suggest different strategies to overcome such difficulties based on the specific characteristics (eg, skill level and personal preferences) of the patient.

It is noteworthy that participants perceiving the treatment as more difficult logged in significantly more, hence, contrary to the classic concept of engagement, greater online interaction does not necessarily mean participants like the treatment or it is useful for them, but instead, that they may be struggling to understand it or to implement the strategies. This defies the traditional concept of adherence in digital interventions, usually determined by the number of logins or intervention modules completed [33]. This emphasizes the importance of assessing online and offline engagement separately, as it seems that youth in our study who were struggling to understand how to follow the treatment needed to engage with the website more, perhaps to review the instructions provided.

From the 4 models proposed, only 1 predicted offline engagement, and with marginal significance. This may be due to other variables not considered in this study (eg, self-efficacy, pain intensity). However, we indeed observed a direct effect on one path of the model: perceiving the treatment as helpful was directly associated with the frequency of use of the skills. Literature is scarce in this area; nevertheless, some studies have shown that the use of the skills taught in digital interventions is a significant mediator in symptom reduction [34], making it a good candidate to be included in future studies on the efficacy of digital psychological interventions.

Future lines of research could use the models presented here to test engagement with digital interventions addressing mental health problems (eg, depression or anxiety) and other health conditions, such as diabetes or asthma.

### Limitations

The findings of this work should be interpreted in light of the following limitations. First, the sample size, although usual for this type of trial, did not allow for subgroup testing or to integrate all the variables of interest in a single, more comprehensive, model. This would have allowed, for example, to test for moderated mediation and to discern the relationship between predictors and mediators. Second, most of the variables were self-reported, which might have led to a reporting bias effect. Finally, the participants lacked racial and ethnic diversity, and most were from a medium-to-high socioeconomic class, limiting generalizability to more diverse groups. Additionally, participants were mostly female, and whereas this is representative of the population with chronic pain, it might limit the generalizability to males.

In spite of the limitations, this study presents some strengths. First, the consideration of online and offline engagement separately is novel and of interest for future research on digital health. Second, the use of a digital intervention whose effectiveness has been well established, and linked to the level of engagement of the participants [21] allows us to develop firmer conclusions on the role of the different variables. Finally, the longitudinal nature of the design provided the opportunity to observe the temporal effects of the predictors on the mediators, the mediators on the outcomes, and the predictors on the outcomes.

### Conclusions

In conclusion, both of the studied baseline characteristics (treatment expectancies and readiness to change) and treatment perceptions (helpfulness and difficulty) had different degrees of direct and indirect effects on both online and offline engagement with a digital psychological intervention. Assessing these variables at baseline and midtreatment may help to determine the risk of nonadherence. Future research should include larger samples to allow for the testing of all the variables in a single model.

## Abbreviations

PSOCQ-A: Pain Stages of Change Questionnaire-Adolescent version
REDCap: Research Electronic Data Capture
ULCI: upper limit 95% confidence interval
WebMAP: Web-based Management of Adolescent Pain
